# Deregulated expression of the 14q32 miRNA cluster in clear cell renal cancer cells

**DOI:** 10.3389/fonc.2023.1048419

**Published:** 2023-04-17

**Authors:** Ravneet Chhabra, Jennifer Guergues, Jessica Wohlfahrt, Stephanie Rockfield, Pamela Espinoza Gonzalez, Shanon Rego, Margaret A. Park, Anders E. Berglund, Stanley M. Stevens, Meera Nanjundan

**Affiliations:** ^1^Department of Cell Biology, Microbiology, and Molecular Biology, University of South Florida, Tampa, FL, United States; ^2^Department of Cell and Molecular Biology, St. Jude Children’s Research Hospital, Memphis, TN, United States; ^3^Department of Gastrointestinal Oncology, H. Lee Moffitt Cancer Center & Research Institute, Tampa, FL, United States; ^4^Department of Biostatistics and Bioinformatics, H. Lee Moffitt Cancer Center & Research Institute, Tampa, FL, United States

**Keywords:** 14q32, miRNA, ccRCC, LPA, iron, DNMT1, claudin-1, ATXN2

## Abstract

Clear cell renal cell carcinomas (ccRCC) are characterized by arm-wide chromosomal alterations. Loss at 14q is associated with disease aggressiveness in ccRCC, which responds poorly to chemotherapeutics. The 14q locus contains one of the largest miRNA clusters in the human genome; however, little is known about the contribution of these miRNAs to ccRCC pathogenesis. In this regard, we investigated the expression pattern of selected miRNAs at the 14q32 locus in TCGA kidney tumors and in ccRCC cell lines. We demonstrated that the miRNA cluster is downregulated in ccRCC (and cell lines) as well as in papillary kidney tumors relative to normal kidney tissues (and primary renal proximal tubule epithelial (RPTEC) cells). We demonstrated that agents modulating expression of DNMT1 (e.g., 5-Aza-deoxycytidine) could modulate 14q32 miRNA expression in ccRCC cell lines. Lysophosphatidic acid (LPA, a lysophospholipid mediator elevated in ccRCC) not only increased labile iron content but also modulated expression of a 14q32 miRNA. Through an overexpression approach targeting a subset of 14q32 miRNAs (specifically at subcluster A: miR-431-5p, miR-432-5p, miR-127-3p, and miR-433-3p) in 769-P cells, we uncovered changes in cellular viability and claudin-1, a tight junction marker. A global proteomic approach was implemented using these miRNA overexpressing cell lines which uncovered ATXN2 as a highly downregulated target. Collectively, these findings support a contribution of miRNAs at 14q32 in ccRCC pathogenesis.

## 1 Introduction

Approximately half a million people globally are diagnosed with renal cell cancers annually (1). Of the numerous subtypes of kidney cancers, the clear cell subtype (ccRCC) comprises >70% of the tumors (1). ccRCC is a metabolic disease characterized by an abundance of intracellular lipid droplets (2, 3). Although disease prognosis is favorable, malignant spread of the disease occurs in >30% of the patients (1). Disease management involves minimally invasive techniques along with nephron sparing methods and surveillance which are efforts to promote quality of life (4). For metastatic disease, targeted chemotherapeutics and immunotherapies are current regimens being utilized (4). Unfortunately, in patients with advanced ccRCC disease, their clinical outcomes remain poor. Therefore, advancements in new treatment regimens and identification of novel biomarkers are direly needed.

One group of molecules that could be considered as clinical targets in ccRCC are microRNAs (miRNAs) (5, 6). These non-coding small RNAs elicit tumor suppressive and oncogenic characteristics by targeting multiple expressed genes in order to alter multiple biological events including cellular proliferation, migration, invasion, epithelial-mesenchymal transition, and cell death events (5, 6). Interestingly, in contrast to eukaryotic mRNAs, the clustering and unified transcription of miRNAs is common (7). These miRNA clusters may be epigenetically regulated in a collective manner; these may be as small as 2 miRNAs as found on chromosome 8, 17, and X or as large as those located on chromosome 14 and 19 (>40 miRNAs) (7). Furthermore, the functional responses elicited by these clustered miRNAs appear to be related (7). Interestingly, ccRCC is characterized by arm level losses at chromosome 3p and 14q. Although the role of *VHL* and other relevant genes at 3p are well established in ccRCC (1), the miRNA cluster present at 14q has been less well studied. However, there are reports of its deregulated expression and potential contributions to the pathogenesis of other cancer types (8, 9).

Herein, we investigate the miRNA expression pattern of selected miRNAs at the 14q32 locus in TCGA kidney tumors and in ccRCC cell lines. We discover that the 14q32 miRNA cluster is markedly downregulated in kidney tumors and cell lines relative to normal kidney tissues and primary renal proximal tubule epithelial (RPTEC) cells, respectively. In particular, the miRNA profile within the 14q32 locus in the cell lines segregates into two subclusters (A and B). In an effort to identify potential modulators of their deregulated expression, we assessed the effects of epigenetic modulators such as agents targeting DNMT1 (e.g., 5-Aza-deoxycytidine), mitogenic lipids (i.e., lysophosphatidic acid (LPA) and sphingosine-1-phosphate (S-1-P) (10–14)), and iron (15, 16). Furthermore, by using an overexpression approach targeting multiple miRNAs at Subcluster A (miR-431-5p, miR-432-5p, miR-127-3p, and miR-433-3p), we identify that these miRNAs can antagonize certain aspects of tumor-like pathogenesis *in vitro*. Collectively, these findings support the contribution of miRNAs at 14q32 in ccRCC and future studies can be designed to further investigate their role and targeting efficacy *in vivo*.

## 2 Materials and methods

### 2.1 Cell culture

As previously reported (17), human renal cancer cell lines of epithelial origin were obtained from ATCC (Manassas, VA) including 786-O and 769-P (both grown in RPMI 1640 containing 8% fetal bovine serum (FBS) and 1% penicillin/streptomycin) as well as A-498 (grown in EMEM containing 8% FBS and 1% penicillin/streptomycin). We also utilized HK-2 cells which are immortalized kidney cells (ATCC). As reported in our earlier published work (17), HK-2 cells were grown in K-SFM basal media containing 50μg/ml bovine pituitary extract and 5ng/ml human recombinant epidermal growth factor. Renal proximal tubule epithelial cells (RPTEC) were obtained from ATCC and cultured using the Renal Epithelial Cell Growth Kit (#PCS-400-04, ATCC) containing 1% penicillin/streptomycin. As previously described (18), fallopian tube secretory epithelial cells (FTSECs), namely FT194 and FT190, were generously provided by Dr. Ronald Drapkin (Department of Obstetrics and Gynecology, University of Pennsylvania, Philadelphia, PA). These cells express Large T Antigen as well as hTERT and were propagated in DMEM:F12 (1:1) containing phenol red and 2% Ultroser G Serum Substitute and 1% penicillin/streptomycin (18). All cell lines were maintained at 37°C with 5% CO_2_; furthermore, they were subjected to regular mycoplasma testing and were identified as negative.

### 2.2 Generation of HA-VHL overexpressing retroviral 769-P cells

HA-tagged wild type *VHL* in a pBABE-puro plasmid was obtained from Addgene (#19234 (19)). HEK 293T cells (p=n+13) were utilized at a density of 1.5 million cells/well in 6-well plates to generate the retrovirion particles. Following cellular adherence, the cells were transfected with a 1:1:1 ratio of pCGP, pVSVG, and the pBABE-puro plasmid (either empty pBABE puro (Addgene #1764 (20)) or *HA-VHL* wild type pBABE-puro) using Fugene HD, similar to that previously described (18, 21). 769-P cells were seeded at 250,000 cells in 6-well plates and infected two times with filtered viruses and polybrene (at 8μg/ml and at 16μg/ml, respectively). Cells were then selected with 0.75μg/ml of puromycin and cultures expanded for validation of HA-VHL protein expression and further analyses.

### 2.3 Immortalization of renal proximal tubule epithelial cells

Primary RPTEC cells were immortalized using a strategy similar to that previously described (18). In our approach, HEK293 cells were seeded at 1.0 x 10^6^ cells/well in 6-well plates and following adherence, the cells were transfected with pCGP and pVSV along with one of the following expression plasmids: empty pBABE-puro (Addgene #1764 (20)) or *SV40 LTAg* (Addgene #14088 (22)) or *TERT* (Addgene #1772 (23)) at a ratio of 1:1:1. Retrovirion particles were collected and 0.45μm filtered at 48 hours post-transfection for use in retroviral infections. After infection, cells were selected in 0.75μg/ml puromycin upon cell culture expansion.

### 2.4 Cell treatments

Using dimethylsulfoxide (DMSO) treated cells as controls, cells were treated with Temsirolimus (TEMS, #50-811-7, Fisher Scientific, Pittsburgh, PA) at a final concentration of 2μM, as described in our prior work (17). Likewise, as earlier described (24), 5-Azacytidine (AZA, #S1782, Selleck Chemicals, Houston, TX) was utilized at a final concentration of 1μM while Suberoylanilide hydroxamic acid (SAHA, #S1047, Selleck Chemicals, Houston, TX) was utilized at a final concentration of 50μM. Lysophosphatidic acid (LPA, as a sodium salt in chloroform, #857130C, Avanti Polar Lipids, Alabaster, AL) was utilized at a final dose of 10μM, as previously described (17). D-erythro-Sphingosine-1-Phosphate (S1P, #860492, Avanti Polar Lipids, Alabaster, AL) was utilized at a final concentration of 250nM. Deferoxamine mesylate (DFO, #D9533, Sigma-Aldrich, St. Louis, MO) was utilized at a final concentration of 10μM, as described in our earlier work (25).

### 2.5 Mimic transfection

769-P cells were seeded at 250,000 cells/well in 6-well plates. Following overnight adherence, cells were next transfected using Fugene HD with the following miRNA mimics: negative control mimic (Ambion, #4464059) or a combination of hsa-miR-433-3p (Ambion, MH10774), hsa-miR-431-5p (Ambion, MH10091), hsa-miR-432-5p (Ambion, MH10941), and hsa-miR-127-3p (Ambion, MH10400). Dissolution of lyophilized mimics were prepared and aliquoted according to the manufacturer’s instructions. Protein lysates and RNA were collected at 48 hours post-transfection.

### 2.6 RNA isolation and quantitative PCR

As previously described (24), RNA was isolated from cells using the miRVana isolation kit (#AM1561, Ambion, NY) or the RNeasy mini kit (QIAGEN, Valencia, CA) following manufacturer’s instructions. RNA was quantified using NanoDrop 1000 (Fisher Scientific, Pittsburg, PA).

Quantitation of gene expression was performed using the TaqMan RNA-to-CT One-Step Kit (#4392938, ThermoFisher Scientific, Waltham, MA) with the following FAM-labelled probes/primers: *CD71* (Hs00951083_m1), *FPN1* (Hs00205888_m1), and *FTH1* (Hs01694011_s1). Normalization of gene expression was performed using Cyclophilin A (PPIA, #Hs04194521_s1, ThermoFisher, Waltham, MA, USA), as previously described (24). miRNA quantitation was also performed using the TaqMan RNA-to-CT One-Step Kit with the following corresponding FAM-labelled probes/primers: miR-493-5p (ID# 001040), miR-431-5p (ID# 001979), miR-432-5p (ID# 001026), miR-411-5p (ID# 001610), miR-495-3p (ID# 001663), miR-539-5p (ID# 001286), miR-323b-3p (ID# 244080_mat), miR-410-3p (ID# 001274), miR-127-3p (ID# 000452), and miR-433-5p (ID# 001028). Normalization of miRNA expression was performed using *RNU48* (ID# 001006). Values were reported as fold change as determined via the 2^-ΔΔCT^ correlative method, as described previously (24).

### 2.7 Protein isolation, SDS-PAGE, and western blotting

As previously described (24, 25), proteins were extracted and normalized to at least 1500μg/ml. Proteins were subsequently analyzed on SDS-PAGE gels (8% or 10%, as appropriate) and transferred to polyvinylidene difluoride (PVDF) membranes for western blotting (24, 25).

Primary antibodies that were utilized in this study are: SV40 LTAg mouse monoclonal (#554149, 1:1000, BD Biosciences), DNMT1 rabbit polyclonal (#5032, 1:1000, Cell Signaling Technology), HA-11 mouse monoclonal (#14921901, 1:500, Covance/Lab Corporation), CD71 mouse monoclonal (#sc-51829, 1:250, Santa Cruz Biotechnology), FTH1 rabbit polyclonal (#3998, 1:500, Cell Signaling Technology), pan-Actin rabbit polyclonal (#4968, 1:1000, Cell Signaling Technology), PARP rabbit polyclonal (#9542, 1:1000, Cell Signaling Technology), PAX-8 rabbit polyclonal (#10336-1-AP, 1:1000, Proteintech), Claudin-1 rabbit polyclonal (#187362, 1:10,000, Invitrogen), BAK rabbit monoclonal (#12105, 1: 1000, Cell Signaling Technology), Bcl-2 rabbit monoclonal (#2570, 1:1000, Cell Signaling Technology), Occludin-1 mouse monoclonal (#611090, 1:250, BD Biosciences), Cyclin D1 rabbit polyclonal (#sc-718, 1:1000, Santa Cruz Biotechnology), and Cyclin E mouse monoclonal (#sc-247, 1:500, Santa Cruz Biotechnology).

### 2.8 Labile iron measurement via FerroOrange assay

Cells were seeded into a 96-well black plate at 1,500 cells/well. After adherence and appropriate treatments, the measurement of iron was performed using the FerroOrange assay kit (Dojindo Molecular Technologies Inc., Rockville, MD), according to manufacturer’s instructions. The supernatant was discarded, and cells were then washed thoroughly with Hank’s Balanced Salt Solution (HBSS) followed by the addition of 1μM of Ferro-Orange (100μl/well) to 100μl of HBSS. The plate was incubated at 37°C for 30 minutes and then read using a Biotek Plate Reader to measure fluorescence intensity at an excitation wavelength of 543nm and an emission wavelength of 580nm.

### 2.9 Cholesterol assay

According to our previously published methodology (17), cholesterol measurements were conducted using the Amplex Red Cholesterol Assay Kit (#A12216, Life Technologies, Grand Island, NY).

### 2.10 Cell viability assay

769-P cells were seeded into 96-well plates at 2,500 cells/well followed by appropriate treatments. The media was discarded and 100μl of crystal violet was added for an incubation period of 15-20 minutes. The dye was then discarded and plates were then washed thoroughly with nanopure water. After overnight drying, 100μl of Sorenson’s buffer was added and plates were incubated for 3 hours at room temperature. The plate was next read using the Biotek Plate reader set at 570nm using the Gen5 software.

### 2.11 Cell migration assay

Samples were processed for migration assay according to manufacturer’s instruction (Cell Biolabs). Transfected cells were seeded into serum-free media into Boyden Chambers at 30,000 cells/insert. The bottom wells were immersed in 500ul of media containing FBS. Cells were incubated overnight (24 hours) prior to washing of inner membrane of insert and staining of migrated cells (bottom of insert) in cell stain solution followed by quantification into extraction solution at 570nm using a Biotek plate reader.

### 2.12 Mass spectrometry-based proteomics and IPA analyses

Samples were processed for mass spectrometry using an iST kit (PreOmics GmbH) per manufacturer’s instructions and reagents, with the slight modification of digesting the sample before loading it onto the cartridge (26). Samples were separated on a nanoElute (Bruker) nanoflow ultra-high performance liquid chromatography (UHPLC) system and analyzed in-line by LC-MS/MS on a trapped ion mobility spectrometry (TIMS)-QTOF instrument (timsTOF Pro, Bruker). A CaptiveSpray ion source with column oven heated to 50°C was utilized for the Aurora series 2 UHPLC reversed-phase C18 column (25 cm × 75 µm i.d., 1.6 µm C18, IonOpticks). Mobile phases A (0.1% formic acid in water) and B (0.1% formic acid in acetonitrile) were used in a 90-minute gradient of 2-25% B, resulting in a total run time of 120 minutes including a ramp up to 37-80% B to clean the column and prepare for the next sample. The timsTOF Pro was set to the default DIA-PASEF scan mode spanning 400-1201 m/z within an ion mobility range of 0.6- 1.43 1/K_0_ [V·s/cm^2^], corresponding to an estimated 1.80 s cycle time. Collision energy and DIA-PASEF windows were 20 eV for a base of 0.85 1/K_0_ [V·s/cm^2^] and 59 eV for 1.30 1/K_0_ [V·s/cm^2^]. Calibration of m/z as well as ion mobility was performed linearly using three ions at 622, 922, and 1222 m/z (Agilent).

DIA data were analyzed in library-free mode [i.e., *in silico* library generated from the Uniprot *Homo Sapiens* database (UP000005640, 78,120 entries)] using DIA-NN (v. 1.8). Label-free quantification (LFQ) with match-between-runs (MBR) with an FDR of 1% was performed using the single pass mode neural network classifier, genes as the protein inference, robust LC (high precision) as the quantification strategy, cross-run normalization that is RT-dependent, and smart profiling library generation.

Statistical testing and bioinformatic analysis were performed in Perseus (v.1.6.15.0) (27) as previously described (28) with some alterations to accommodate DIA-NN results. To summarize, we removed the contaminants from DIA-NN pg.matrix output file and uploaded the remaining 10,232 protein groups (which includes redundancy based on algorithm grouping) to Perseus. LFQ values were then log2 transformed and annotated into separate treatment groups before being filtered 2/3 in at least one group resulting in 10,191 protein groups. Remaining missing values were imputed with a width of 0.3 and a downshift of 1.8 to fit the lower abundance of the Gaussian curve (29). A Welch’s t-test with a p-value cutoff of < 0.05 was then used. To apply hierarchical clustering and gene ontology (GO) annotation in Perseus, the list was filtered for only those statistically significant before being normalized. Hierarchical clustering of two major groups was performed and GO annotation was then added. Cluster enrichment was tested using a Fisher’s exact test with Benjamini-Hochberg (BH) correction at an FDR <0.05. Furthermore, the entire list (before significance filtration) was exported and in addition to Welch’s t-test significance cutoff of <0.05, a Z-score cutoff of >1 was also implemented to increase confidence for subsequent validation (30). The list that utilized Welch’s t-test and Z-score cutoffs was then uploaded to Ingenuity Pathway Analysis (IPA) for bioinformatic analysis. We then added a miRNA list using experimentally measured fold-change values for the overexpressed miRNAs and generated a miRNA target filter to connect this list to our experimentally generated dataset of differentially expressed proteins.

### 2.13 Bioinformatic analyses

The OncoLnc (http://www.oncolnc.org) online tool was used to calculate differences in survival for selected miRs in the KIRC and KIRP cohorts, as compared to all other TCGA cohorts. All significant (FDR-corrected p-values < 0.05) CoxPH coefficients were calculated for each miRNA in each TCGA cohort using the OncoLnc tool. Kaplan survival curves were then drawn using either the top/bottom 15% (KIRC) or top/bottom 25% (KIRP) patients based on miRNA expression values.

miRNA expression data (expressed as Log_2_ CPM) for both the KIRC and KIRP cohorts were downloaded from CancerMIRNome (http://bioinfo.jialab-ucr.org/CancerMIRNome/). Datasets were filtered to the miRNAs in the 14q32 miRNA cluster shown in **Supplemental Table 1**. Heatmaps were generated using R version 4.0.5 and the heatmap.2 function.

Raw IDAT files for KIRP and KIRC were downloaded and normalized using NOOB (31). β-values with a corresponding detection p-value > 0.01 were set to missing values. 293 tumors samples classified as ccRCC were based on the re-classification by Ricketts et al. (32) together with 205 normal kidney samples. Masked CpG-probes were removed based on the recommendation by Zhou et al. (33) and the hg38-based re-annotation was downloaded from http://zwdzwd.github.io/InfiniumAnnotation. The differential methylation was calculated between normal kidney and ccRCC tumors using a two-sided Students t-test assuming unequal variance. Multiple testing (FDR) was adjusted using the method by Benjamini and Hochberg (34). Δ β-value was calculated by comparing the average β-value for the normal and the tumor samples. The methylation analysis and visualization were done in MATLAB 9.13.0 (R2022b), Natick, Massachusetts: The MathWorks Inc.

### 2.14 Statistical analyses

Experiments were performed at least in three independent replicates, unless otherwise described. GraphPad version 6.04 Prism software (GraphPad, La Jolla, CA) was utilized to generate statistical analyses wherein *p-*values were produced using the nonparametric Student’s *t-*test and error bars shown are represented as the average with standard deviations, unless otherwise indicated. NS represents values of non-significance; *p-values of ≤ 0.05; **p-values ≤ 0.01; ***p-values ≤ 0.001; and ****p-values ≤ 0.0001.

## 3 Results

### 3.1 Reduced expression of the 14q32 miRNA cluster in clear cell and papillary kidney tumors

**Figure 1:**
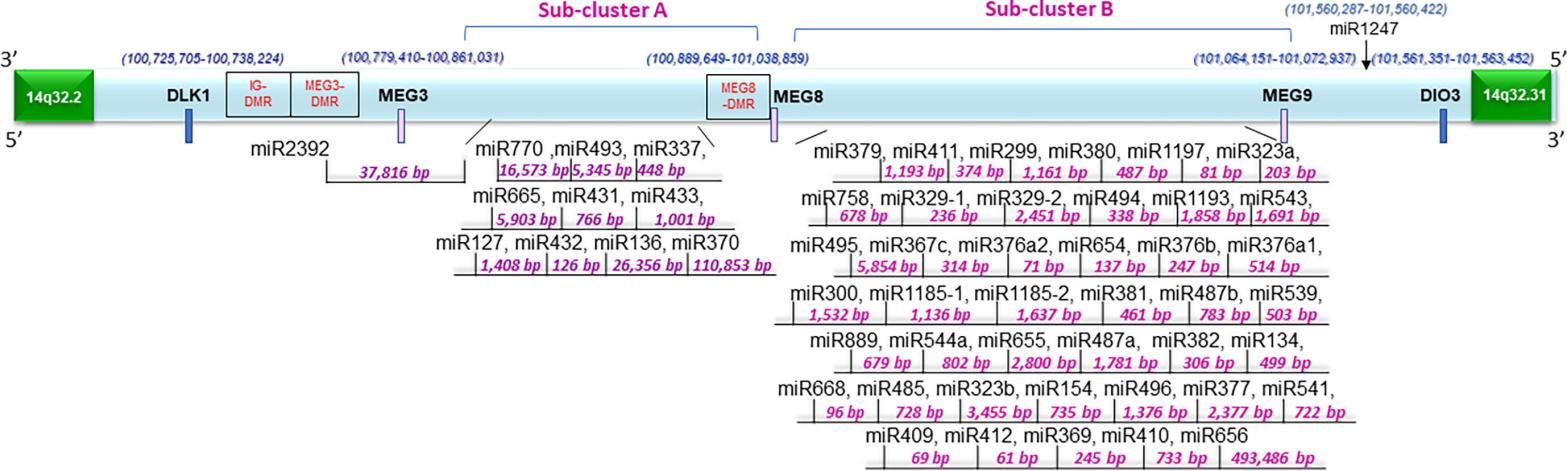
Schematic representation of the 14q32 miRNA cluster and surrounding region. Fifty-four miRNAs are located at the region depicted in two physically distinct subclusters. There are differentially methylated regions at this locus annotated as IG-DMR, MEG3-DMR, and MEG8-DMR. The distances between miRNAs were determined based on data derived from the UCSC genome browser (Dec. 2013 (GRCh38/hg38)) (36).

**Figure 2:**
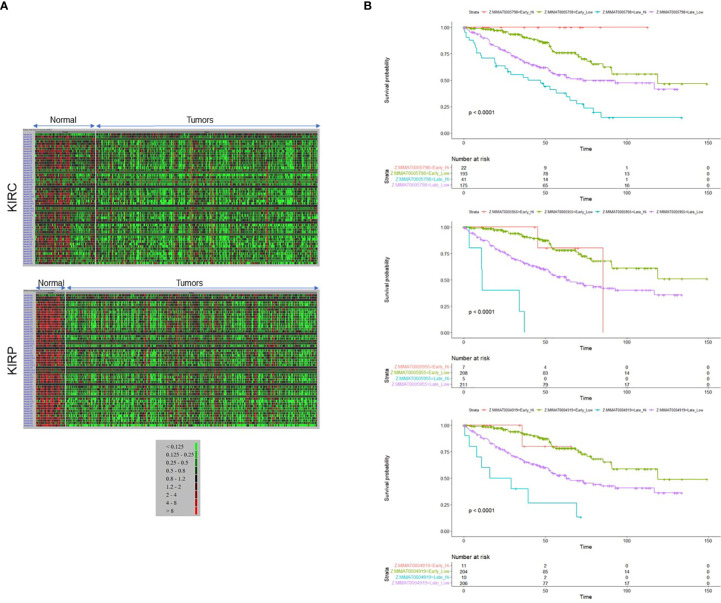
14q32 microRNA expression level corresponds to the disease aggressiveness. **(A)** Heat maps representing 14q32 miRNAs generated from TCGA patient data derived from **(A)** KIRC (top panel) and KIRP (bottom panel) data sets. **(B)** Patient survival outcomes for MIMAT0005798 (miR-1185-5p), MIMAT0005955 (miR-1197), MIMAT0004919 (miR-541-5p) are shown.

Along with the most common arm-level genomic aberrations associated with ccRCC that is loss of *VHL* at chromosome 3p (an obligate event (1)), there exist other regions that are aberrant including gains at chromosome 5p and loss at chromosome 14q (35). Loss of 14q is a common event in patients with aggressive ccRCC tumors (35); however, its role and contribution to ccRCC pathogenesis is lesser studied. Upon close examination of the 14q arm commonly deleted in ccRCC patients, one of the largest miRNA clusters in the human genome is noted at 14q32, composed of 54 miRNAs (**Figure 1**). The miRNAs at 14q32 are separated into two subclusters by MEG8 which separates subcluster A (miR-770 to miR-370) from subcluster B (mR-379 to miR-656) (37). To investigate alterations in the expression of the 14q32 miRNAs in kidney cancer patients, The Cancer Genome Atlas (TCGA) kidney tumor data sets, namely KIRC (clear cell) and KIRP (papillary) (38, 39), were analyzed. Expression data for both the KIRC and KIRP cohorts were downloaded from CancerMIRNome (http://bioinfo.jialab-ucr.org/CancerMIRNome/). Sample types were either tumor tissue (n=516 for KIRC and n=291 for KIRP) or adjacent normal (n=71 for KIRC and n=34 for KIRP). Heatmaps were generated for normal tissues (left) and tumor tissues (right side) in **Figure 2A** using 14q32 miRNAs listed in **Supplemental Table 1**. As shown in **Figure 2A** (median-fold change from normal and FDR adjusted p-values (q-values) for each miRNA are presented in **Supplemental File 1**), the majority of the 14q32 miRNAs have decreased expression in tumor samples in both KIRC and KIRP, as compared to controls (adjacent normal tissues from individuals in the TCGA cohort).

As shown in **Figure 2B**, survival was filtered for Stage II-IV patients (equivalent to “late stage”) versus Stage I (equivalent to “early stage”). We noted a maximum survival of approximately 6 years for some patients with miRNA “high-expressors”. When restricting to patients with a survival time of ≤ 5 years, there were very few patients and thus the survival data was not restricted in this manner for statistical analysis. We have stratified patients in the KIRC cohort into “high-expressors” and “low-expressors” based on a mean split (patients with z-scores ≤ mean were stratified into “low expressors” and patients with z-scores > mean were stratified into “high-expressors”), since most patients did not express high levels of miRNAs in this cluster. Since few patients expressed high miRNA levels in the Stage I cohort, we performed an analysis on a subset of cluster-specific miRNAs that includes stage I while categorizing patients into 4 cohorts: early stage “low-expressors”, early stage “high-expressors”, late stage “high-expressors”, and late stage “low-expressors”. Interestingly, the findings from these analyses suggest that the patients’ survival in early stage is not significantly affected by miRNA expression levels for most of the cluster miRNAs (see **Supplemental File 2** for p-values, p-adjusted, and HR values and see **Supplemental File 3** for CI values for Kaplan Meier analyses).

### 3.2 Reduced expression of the 14q32 miRNA cluster in clear cell renal cancer cell lines

**Figure 3:**
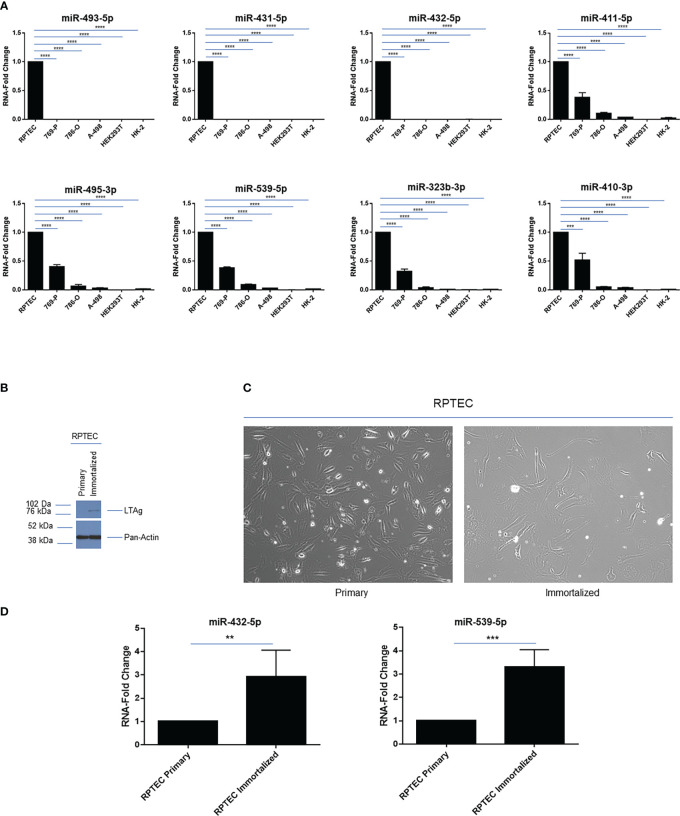
14q32 miRNA expression across a series of renal cell lines. **(A)** qPCR analysis of selected 14q32 miRNAs across 6 renal cell lines. miRNA-fold change values are displayed for two independent replicates (each measured in triplicate) using *RNU48* as a reference control. **(B)** Protein lysates were obtained from primary and immortalized RPTEC cells followed by western blotting using the antibodies shown. **(C)** Representative images of primary and immortalized RPTEC cells are shown which were captured at 20x magnification. **(D)** qPCR analyses of miR-432-5p and miR-539-5p in primary and immortalized RPTEC cells. miRNA-fold change values are displayed using *RNU48* as a reference control. ** *p*-values ≤ 0.01; *** *p*-values ≤ 0.001; **** *p*-values ≤ 0.0001.

We next investigated the expression pattern of a subset of miRNAs within the 14q32 cluster in a series of kidney cell lines. These include those that are cancer-derived from clear cell renal cell tumors, namely 769-P, 786-O and A-498, which we previously reported to have increasing number of copy number alterations (17), primary renal proximal tubule cells (RPTEC), and immortalized cell lines commonly utilized in the field of kidney biology such as HEK293T and HK-2. As shown in **Figure 3A**, we focused on selected 14q32 miRNAs within subcluster A (miR-493-5p, miR-431-5p, and miR-432-5p) and B (miR-411-5p, miR-495-3p, miR-539-5p, miR-323b-3p, miR-410-3p) and analyzed their expression levels via real-time PCR using total RNA. As expected, all of the miRNAs selected for this analysis displayed robust miRNA expression level in RPTEC cells. In contrast, all 14q32 miRNAs were reduced in the three selected ccRCC cell lines (normalized to *RNU48*). The pattern of expression was similar within each subcluster, suggesting that the miRNAs located within these two independent regions may be independently micro-regulated.

However, the two immortalized cell lines, HEK293T and HK-2, also had low expression of these 14q32 miRNAs, implicating the contribution of the immortalization process and/or other events to the reduced miRNA expression at the 14q32 locus in these cells. This data indicates that these immortalized cell lines are not appropriate cellular models for investigating the 14q32 miRNA cluster in ccRCC studies.

To determine whether the immortalization process may contribute to an altered miRNA expression profile, we retrovirally infected primary RPTEC cells with *SV40 LTAg* and *TERT*. Following cell selection with puromycin, we assessed and validated the protein expression level of Large T Antigen (LTAg) (**Figure 3B**); however, TERT was not detectable via western blot analysis. Although the immortalized RPTEC cells displayed changes in morphology (more elongated) compared to the primary RPTEC cells (**Figure 3C**), the miRNA analysis did not show any marked reductions (rather an increase was noted) in miR-432-5p (within subcluster A) and miR-539-5p (within subcluster B) expression (**Figure 3D**). Although these findings indicate that LTAg does not reduce miRNA expression under our experimental conditions, different immortalization processes might have a different effect on miRNA expression in the 14q32 locus since HEK293T cells and HK-2 cells were immortalized in a different manner, namely using human papilloma virus 16 (HPV-16) *E6/E7* genes and Ad5 *E1A* and *E1B*, respectively (40, 41).

### 3.3 Epigenetic regulation of 14q32 miRNAs in ccRCC cell lines

In ccRCC, the combination of epigenetic alterations along with clinical variables can identify ccRCC patients who are at elevated risk for aggressive disease (42). Such epigenetic alterations are expected to drive the loss of expression of genes and non-coding RNAs. As shown in **Figure 1**, the 14q32 region contains three differentially methylated regions (i.e., IG-DMR, MEG3-DMR, and MEG8-DMR) which may contribute to the regulation of the 14q32 miRNA cluster (37).

**Figure 4:**
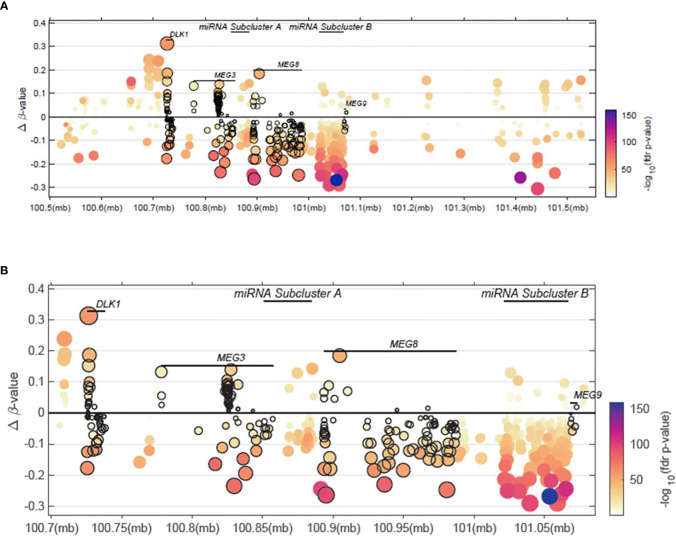
Methylation patterns within the 14q32 region of interest. **(A)** Delta β-values across a 14q32 region encompassing <100.6MB to >101.5MB is shown. **(B)** Delta β-values from a 14q32 region focusing on <100.75MB to >101.05MB is shown, spanning the *DLK1*, *MEG3*, miRNA subcluster A, *MEG8*, and miRNA subcluster B regions.

In an effort to analyze available high-throughput profiling data of DNA methylation status of CpG islands across our region of interest at 14q32, we analyzed TCGA methylation data for this region. As shown in **Figure 4** and **Supplementary File 4**, we note that there is both hyper- and hypo- methylation across this region of interest across *DLK1* through *MEG9* in tumors samples, compared to normal tissue. The highest levels of hyper-methylation across ~100MB through 101.5MB appears to occur at the DLK1 locus, followed by MEG8 and MEG3. β-values are presented as an approximation of the methylation percentage at the sites shown along with –log_10_ FDR p-values.

**Figure 5:**
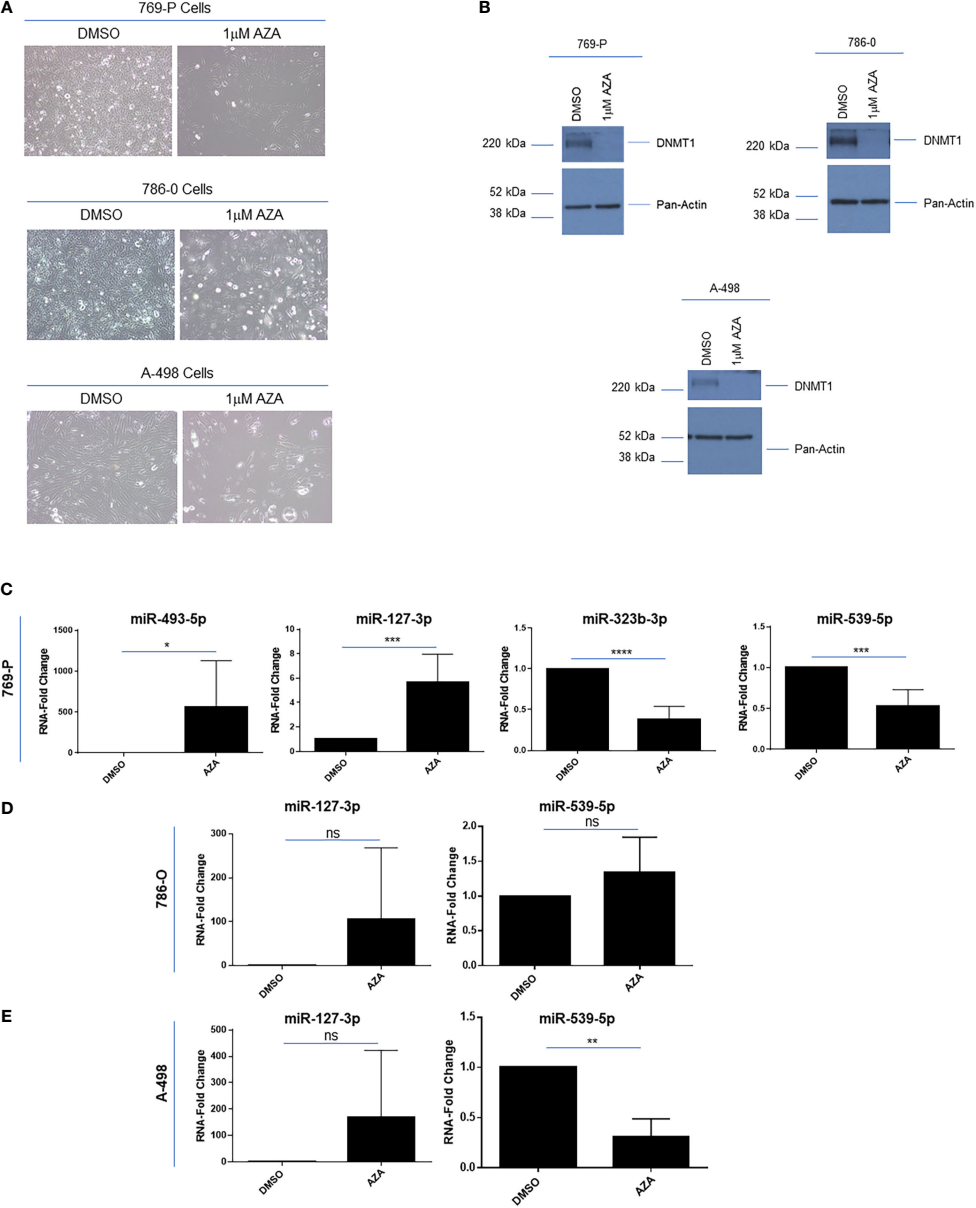
Epigenetic regulation of 14q32 miRNAs in ccRCC cell lines. **(A)** Light microscopic images of 769-P, 786-O, and A-498 cells treated with 1μM Azacytidine (AZA) for 120 hours at 20x magnification. **(B)** Protein lysates were obtained from AZA-treated ccRCC cells followed by western blotting using the antibodies shown. **(C)** qPCR analyses of miR-493-5p, miR-127-3p, miR-323b-3p, and miR-539-5p (normalized to RNU48) in DMSO- and AZA-treated 769-P cells. The data represents a composite of three independent replicates. **(D)** qPCR analyses of miR-127-3p and miR-539-5p in DMSO- and AZA-treated 786-O cells. The data represents a composite of three independent replicates. **(E)** qPCR analyses of miR-127-3p and miR-539-5p (normalized to RNU48) in DMSO- and AZA-treated A-498 cells. The data represents a composite of three independent replicates. ns, not significant; * *p*-values ≤ 0.05; ** *p*-values ≤ 0.01; *** *p*-values ≤ 0.001; **** *p*-values ≤ 0.0001.

In melanoma cell lines, the downregulated expression of these 14q32 miRNAs was shown to be re-activated by cellular treatments with demethylating drugs and/or histone deacetylase inhibitors (43). To determine whether the miRNAs within the 14q32 cluster could be re-expressed following methylation inhibition in ccRCC cells, we treated 769-P, 786-O and A-498 cells with 1μM 5’-Azacytidine (AZA). As shown in **Figure 5A**, we noted that the cell numbers were markedly reduced along with a change in morphology, following 120 hours of treatment. The cellular response to AZA was validated via western analyses for DNA methyltransferase 1 (DNMT1), which was reduced in its expression following cellular AZA treatment (**Figure 5B**). Interestingly, we noted that the expression of miRNAs from subcluster A were increased 565-fold (*p* ≤ 0.05) for miR-493-5p and 5.7-fold (*p* ≤ 0.001) for miR-127-3p, while the subcluster B miRNAs (miR-323b-3p and miR-539-5p) were reduced (47% (*p* ≤ 0.001) and 62% (*p* ≤ 0.0001), respectively) in 769-P cells (**Figure 5C**). Although similar trends were observed in 786-O and A-498 for miR-127-3p, reduced miR-539-5p (at 69%, *p* ≤ 0.01) was noted in A-498 cells (**Figures 5D, E**). Studies were also performed with SAHA, a histone deacetylase inhibitor, without any marked alterations in miRNA expression in 769-P cells (results not shown). Altogether, these findings suggest that the miRNAs within the two subclusters may be differentially methylated in renal tumor cell lines and therefore the two clusters within the 14q32 locus may be subjected to differential micro-regulation.

### 3.4 The lysophospholipid mediators, lysophosphatidic acid and sphingosine-1-phosphate, modulate 14q32 miRNA expression in primary RPTECs

Amongst established mediators of epigenetic regulation are the lysophospholipids (lysophosphatidic acid (LPA) and sphingosine-1-phosphate (S1P)). For example, during the process of oligodendrocyte differentiation, the ATX-LPA pathway was identified to alter histone deacetylase (HDAC) activity and consequently gene expression (14). Further, LPA was also shown to contribute to cancer cell survival by elevating HDAC activity (10). On the other hand, nuclear localized S1P inhibits HDAC activities in which sphingosine kinase 2 (SPHK2) bound to HDAC1/2 are enriched at promoter elements (44). LPA is a mitogenic lipid that is generated via the activity of lysophospholipase D (also called autotaxin (ATX or ENPP2)) using lysophosphatidylcholine (LPC) as the substrate. LPA is present in multiple biological fluids and is elevated in variety of tumors as a result of elevated ATX expression (45). Of relevance to renal cancer, ATX is significantly increased in kidney tumors (45, 46). Likewise, sphingosine kinase 1 (SK1) is elevated in ccRCC cell lines along with elevated S1P levels, a related lysophospholipid mediator (47).

**Figure 6:**
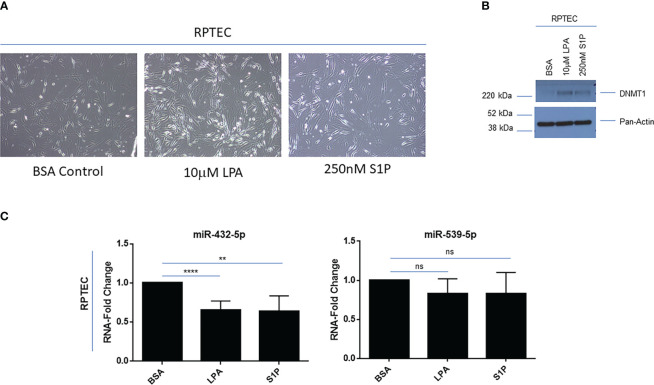
Lysophosphatidic acid (LPA) and sphingosine-1-phosphate (S1P) modulate 14q32 miRNA expression in primary RPTECs. **(A)** Light microscopic images of RPTEC cells treated with BSA, LPA, or S1P captured at 20x magnification. **(B)** Protein lysates were obtained from BSA, LPA, or S1P treated RPTEC cells followed by western blotting using the antibodies shown. **(C)** qPCR analyses of miR-432-5p and miR-539-5p (normalized to *RNU48*) in RPTEC in response to treatment with BSA (control), LPA, or S1P. The composite of three independent experiments is shown. ns, not significant; ** *p*-values ≤ 0.01; **** *p*-values ≤ 0.0001.

Due to these renal cancer specific findings, we next investigated whether LPA or S1P treatment in primary RPTEC cells could modulate 14q32 miRNA expression. Upon long-term LPA (10μM) or S1P (250nM) treatment up to 9 days, there was a marked alteration in the morphology of RPTEC cells, similar to features of epithelial-mesenchymal transition (EMT) (**Figure 6A**), which was accompanied by increased expression of DNMT1 as shown via western blotting (**Figure 6B**). With respect to 14q32 miRNA expression, miR-432-5p (located within subcluster A) was reduced (35% (miR-432-5p with LPA), 37% (miR-432-5p with S1P) with trends in reduced levels for miR-539-5p (located within subcluster B) following lysophospholipid and S1P treatment (**Figure 6C**). These findings suggest that the miRNA changes may contribute to lysophospholipid induced kidney cancer pathogenesis. In support, reductions in miR-539-5p expression have been reported to contribute to kidney cancer progression (48).

### 3.5 Iron-dependent regulation of miR-410-3p, a 14q32 subcluster B miRNA

**Figure 7:**
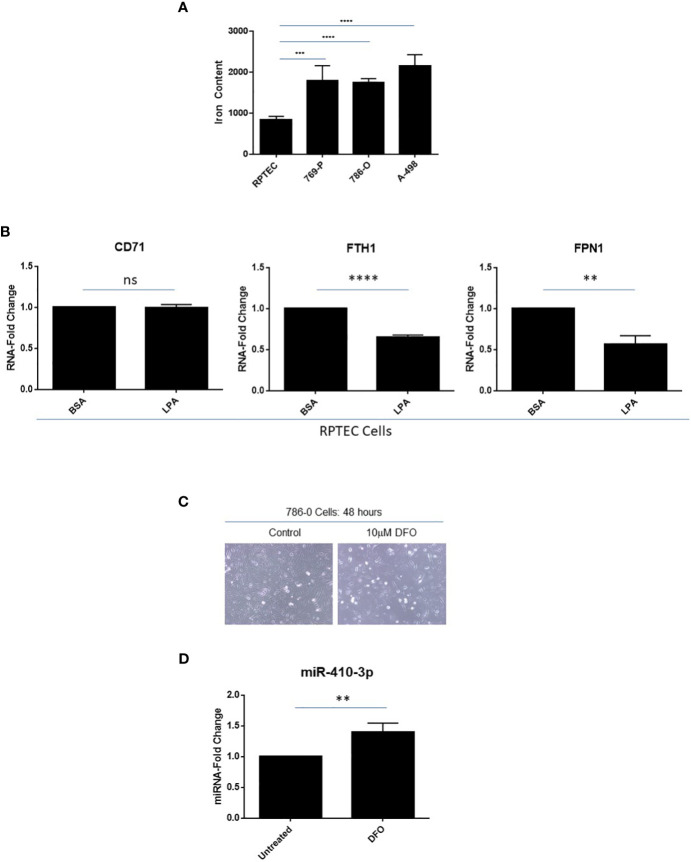
Elevated labile iron content in ccRCC with iron-dependent regulation of miR-410-3p, a 14q32 miRNA, in 786-O cells. **(A)** Labile iron was measured using FerroOrange in RPTEC, 769-P, 786-O, and A-498 cell lines. **(B)** qPCR analyses of *CD71*, *FTH1*, and *FPN1* (normalized to *PP1A*) in RPTEC in response to treatment with BSA (control) or LPA. The composite of three independent experiments is shown. **(C)** Light microscopic images of 786-O cells treated with 10μM DFO for 48 hours were captured at 20x magnification. **(D)** qPCR analyses of miR-410-3p (normalized to *RNU48*) in untreated and DFO-treated 786-O cells. ns, not significant; ** *p*-values ≤ 0.01; *** *p*-values ≤ 0.001; **** *p*-values ≤ 0.0001.

In multiple cancer types, the intracellular labile iron content is elevated thus contributing to their increased growth, proliferation, and metastatic propensity (49); indeed, tumors have been described as “addicted” to iron (50). In addition to elevated transferrin receptor expression in clinical ccRCC specimens (51), we show that intracellular iron levels are also elevated in ccRCC cell lines including 769-P (2.1-fold, *p* ≤ 0.001), 786-O (2.0-fold, *p* ≤ 0.0001), and A-498 (2.6-fold, *p* ≤ 0.0001) cells relative to primary RPTEC cells (**Figure 7A**). Interestingly, as shown in **Figure 7B**, we noted that LPA treatment of RPTEC cells resulted in significantly reduced RNA expression in mediators of the iron metabolic pathway including ferritin heavy chain (*FTH1*, 35%, *p* ≤ 0.0001) and the iron exporter channel, ferroportin (*FPN1*, 43%, *p* ≤ 0.01). These molecular alterations mediated by LPA suggest that there is a likely resultant increase in intracellular labile iron, which may contribute to the LPA-induced tumorigenic response. In support, as shown in **Supplemental Figure 1**, LPA treatment of epithelial cells (i.e., fallopian tube secretory epithelial cells, FTSECs (18)) leads to altered iron metabolic markers, which may contribute to observed functional responses; however, further investigations need to be performed to clarify the underlying mechanism.

Loss of *VHL* (located at chromosome 3p25) is a genomic event observed in >90% of ccRCC patients and is associated with elevated intracellular iron content which may be a critical event in supporting RCC tumorigenesis (52). To investigate whether altered expression of VHL may lead to altered miRNA expression at 14q32, we overexpressed HA-tagged VHL in 769-P cells (**Supplemental Figure 2**). Generation of stably expressed VHL was confirmed by validating HA expression via western blotting analyses. Both *FTH1* and *FPN1* RNA levels were both significantly elevated (both at 1.4-fold, *p* ≤ 0.05 and *p* ≤ 0.01, respectively); a subtle increase at the FTH1 protein level was also noted. However, there was no consistent change in selected 14q32 miRNA expression implicating other events may be required to reverse the effect on iron response following *VHL* loss (results not shown). Further, when we exposed 786-O cells to Deferoxamine (DFO), an iron chelator, not only were lower cell numbers observed (**Figure 7C**) but miR-410-3p levels (subcluster B miRNA) were significantly increased (1.4-fold, *p* ≤ 0.01, **Figure 7D**). miR-410-3p was selected based on an earlier report of its expression being altered following iron chelation (53). This result supports iron-mediated regulation of miR-410-3p in the 14q32 locus in 786-O cells, although the detailed mechanism underlying this observation needs to be further explored.

### 3.6 Subcluster A miRNAs reduce cellular viability and intracellular labile iron while increasing claudin-1 protein

**Figure 8:**
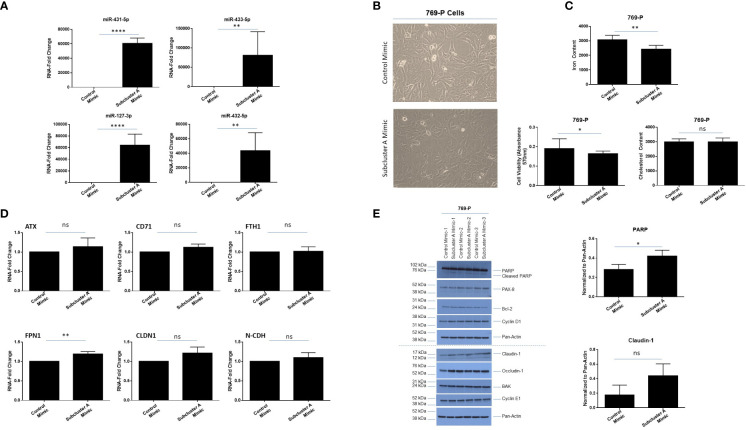
Subcluster A mimic transfection modulates labile iron content and claudin-1 expression in 769-P cells. **(A)** qPCR analyses of miR-431-5p, miR-433-5p, miR-127-3p, and miR-432-5p, and RNU48 in 769-P cells. **(B)** Representative microscopic images of 769-P cells at 48 hours post-transfection with either control mimic or subcluster A mimic at 20x magnification. **(C)** Measurement of the labile iron content (top panel), cell viability via crystal violet (bottom left panel), and cholesterol content (bottom right panel) following miRNA transfection in 769-P cells at 48 hours. The composite of three independent experiments is shown. **(D)** qPCR analyses of *ATX*, *CD71*, *FTH1*, *FPN1*, *CLDN1*, and *N-CDH* are shown, normalized to *PP1A*. The data represents composite of three independent experiments. **(E)** Protein lysates were obtained from control or subcluster A mimic transfected 769-P cells at 48 hours post-transfection. Western blotting using the antibodies shown was then performed. Densitometric analyses for PARP and claudin-1 are shown in the right panels. ns, not significant; * *p*-values ≤ 0.05;** *p*-values ≤ 0.01; **** *p*-values ≤ 0.0001.

In order to investigate the effect of overexpressing miRNAs at 14q32 locus, we co-expressed four miRNAs within subcluster A, namely miR-127-3p, miR-431-5p, miR-432-5p, and miR-433-3p. As shown in **Figure 8A**, qPCR analyses confirmed increased expression of these specific miRNAs at 48 hours post-transfection in 769-P cells. Coinciding with a notable change in morphology (larger and flatter cells), the cellular viability (as assessed via crystal violet staining) was reduced by 14% (*p* ≤ 0.05, **Figures 8B**, **C**, bottom left panel). Cholesterol content was also measured as it is associated with tumorigenic responses in clear cell renal cancer cell lines (54); however, cholesterol levels remained unchanged following miRNA overexpression (**Figure 8C**, bottom right panel). In contrast, we noted a reduction of 22% (*p* ≤ 0.01) in intracellular labile iron content (**Figure 8C**, top panel), which was supported by elevated FPN1 RNA expression (1.2-fold (*p* ≤ 0.01), **Figure 8D**). These findings suggest that the miRNA expression may promote iron export to mediate reduction in intracellular iron content in 769-P cells.

Since we noted a marked change in morphology following expression of subcluster A miRNAs, we examined expression of markers associated with cell death and epithelial-mesenchymal transition (EMT). As shown in **Figure 8E**, we noted elevated cleaved PARP (1.5-fold, *p* ≤ 0.05, a marker of apoptosis) in the absence of changes in PAX8, Bcl-2, BAK, Cyclin D1, or Cyclin E1. Furthermore, although mRNA levels of claudin-1 were not significantly elevated (only an increased trend, **Figure 8D**), we identified a 2.5-fold increase in claudin-1 protein via western blotting analysis (**Figure 8E**), in contrast to occludin-1 which remained unchanged. In support, claudin-1 expression has been reported to be reduced in ccRCC (55). We note from proteomic analysis (described below in section 3.7) that claudin-1 upregulation was observed as a trend (1.5-fold change, p=0.0692) while PAX8 demonstrated subtle downregulation (-1.26-fold change, p=0.002), highlighting the complementary nature of these approaches related to differences in quantitation precision and sensitivity. However, further measurement of EMT markers via real-time PCR (e.g., *SNAI1*, *SNAI2*, *N-CDH*, *TWIST*) (**Supplemental Figure 3**) or western analyses (N-CDH and vimentin, results not shown) did not uncover changes upon overexpression of subcluster A mimics. Additionally, there was no significant difference in migration via use of Boyden Chambers between cells transfected with subcluster A and control mimic (**Supplementary Figure 3**). Altogether, these results suggest that miRNAs within subcluster A may contribute to reduced viability as well as potential alterations in tight junction and/or adhesion function (56).

### 3.7 Proteomic profiling of overexpressed subcluster A miRNAs in 769-P cells identifies ATXN-2 as a top target

**Figure 9:**
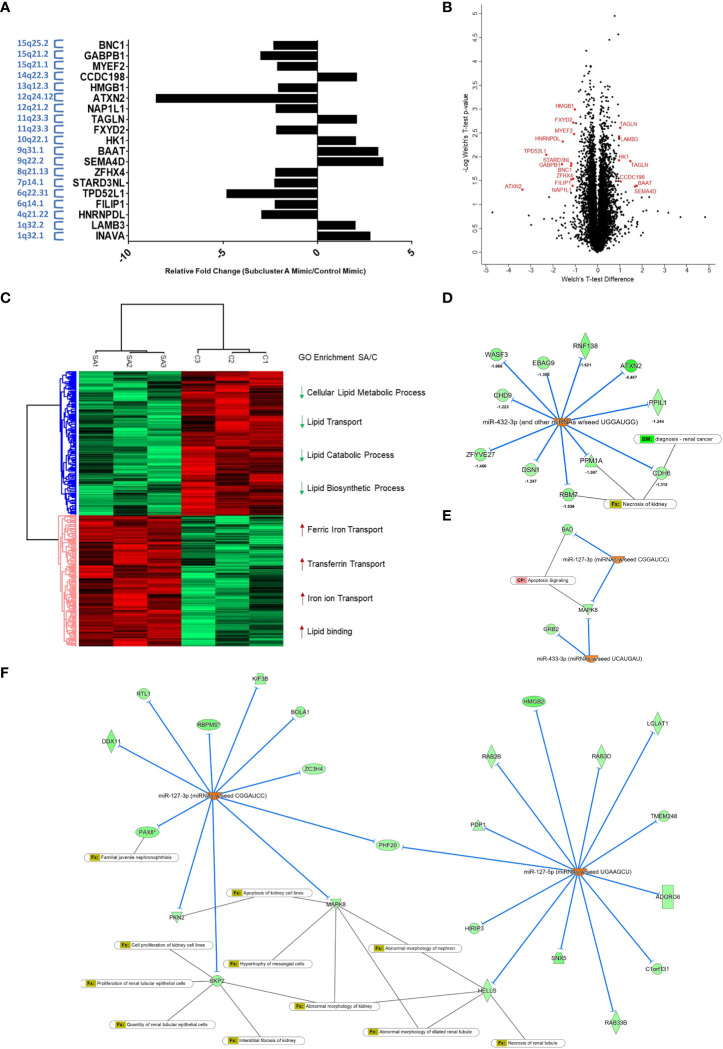
Global proteomic profiling of subcluster A miRNA expression in 769-P cells identifies ATXN-2 as a downregulated target. **(A)** Bar graph representation of 19 targets for miRNA subcluster overexpressed cells with p<0.05 and Z-score >1. The chromosomal locations are indicated with the y-axis labels. **(B)** Volcano plot demonstrating subcluster A miRNA-induced differentially expressed proteins with proteins highlighted in red representing additional filtering related to fold-change (Welch’s t-test, p<0.05 and Z-score>3). **(C)** Hierarchical clustering showing two major groups and related cluster-specific gene ontology enrichment results for selected biologically relevant pathways (Fisher’s Exact test with a Benjamini-Hochberg FDR<0.05). **(D)** Analyses *via* Ingenuity Pathway Analysis was subsequently performed to identify miRNA and proteome dataset relationships. Upregulation of miR-432-3p is predicted to affect the experimentally observed downregulation of several proteins, which included the highly downregulated target ATXN-2, as well as other targets that overlap with renal cancer biomarkers (CDH6) and necrosis of kidney (CDH6 and RBM7). **(E)** The downstream effect on apoptosis signaling was also a predicted result of decreased expression of BAD and MAPK8, which are targets of miR-127-3p and miR-433-3p. **(F)** Upregulated miR-127-3p and miR-127-5p are predicted to downregulate several experimentally observed proteins identified from proteomic analysis, which are associated with pathophysiological processes of the kidney.

In order to expand our understanding of the ccRCC cellular response to re-expression of subcluster A miRNAs in 769-P cells, we utilized a global mass spectrometry-based proteomics approach. We compared the global proteomic profile in cells with subcluster A miRNA (i.e., miR-127-3p, miR-431-5p, miR-432-5p, and miR-433-3p) relative to control mimic transfected cells and identified 8110 ± 58 unique proteins per run. The final list (expanded based on DIA-NN grouping, resulting in 10232 identifications) was then filtered using a Welch’s t-test p-value cutoff of <0.05 leading to 1382 differentially expressed proteins. Furthermore, another filter was applied in which a Z-score cutoff of >1 was added, leading to 630 differentially expressed proteins. The %CV of the subcluster A mimic group was 6.43% median and 9.34% average while the control group was 8.69% median and 12.2% average. To determine the topmost differentially expressed proteins, we identified those with an LFQ intensity ratio of ≥2 or ≤0.5 (corresponding to a more stringent |Z-score| > 3) resulting in 19 identified targets for miRNA subcluster overexpressed cells (**Figure 9A** and **Supplemental Table 2**). The Volcano plot, as shown in **Figure 9B**, highlights these proteins of interest from the statistically significant group. In addition, hierarchical clustering (**Figure 9C**) displays two main clusters corresponding to 1380 and 1370 up- and down-regulated proteins (Welch’s t-test, p < 0.05), respectively. GO enrichment analysis reveals significant depletion of lipid transport and lipid metabolic processes within the downregulated protein cluster and significant enrichment of iron transport processes that are induced by overexpression of the subset of 14q32 miRNAs. The list of total quantifiable proteins, with differentially expressed proteins highlighted, are provided in **Supplemental Table 3**. Proteomic analysis identified ATXN-2 as the target most altered with a fold change of -8.49 in the subcluster A miRNA overexpression group compared to control. Analyses via Ingenuity Pathway Analysis was subsequently performed to identify protein targets as well as relevant canonical pathways that are affected by overexpression of the four subcluster A miRNAs. This analysis resulted in the prediction of miR-432-3p upregulation to influence (i.e., downregulate) several experimentally observed proteins including ATXN2, CDH6, and RBM7, the latter two being closely associated with necrosis of kidney (**Figure 9D**). Interestingly, predicted downstream effects also included apoptosis signaling as a result of decreased expression of BAD and MAPK8 (**Figure 9E**), which was predicted to be associated with miRNA-127-3p and miRNA-433-3p upregulation. The broad range of renal effects based on overexpression of these subcluster A miRNAs can also be observed using miR-127 (3p and 5p) as an example, which is predicted to be an upstream regulator of the experimentally observed downregulated proteins that are associated with abnormalities of kidney processes (**Figure 9F**).

## 4 Discussion

ccRCC is characterized by arm-wide chromosomal alterations. Herein, we demonstrate that there is significant downregulation in expression of miRNAs at 14q32 in ccRCC. The cluster is comprised of 54 miRNAs and represents one of the largest miRNA clusters in the human genome (9). Moreover, it is segregated into two parts, in which the miR-379/miR-656 spans ~45kb and consists of 42 miRNAs (9). The 14q32 locus (between *DLK1* and *DIO3*) contains three differentially methylated regions (DMRs) which may be responsible for regulating expression of the miRNA cluster at 14q32; it has been suggested that these epigenetic alterations may drive the loss of expression of genes and miRNAs within this genomic locus (9, 57). Indeed, based on inhibition of DNA methyltransferases via AZA, one potential mechanism underlying the 14q32 miRNA deregulation is epigenetic modification of regulatory regions at this locus. In addition to deregulated methylation at DMRs, it is noted that the 14q32 locus (such as in osteosarcoma) contains an enrichment of histone modifications (i.e., H3K9-me2, H3K4-me3 and H3K27-me3), as an imprinting defect (57). However, we did not identify any re-expression of miRNAs at 14q32 following histone deacetylase inhibition with SAHA under our experimental conditions in the ccRCC cell lines investigated (results not shown). Additional means of modifying miRNA expression may include those relating to biogenesis and maturation of miRNAs, which requires further detailed investigations. Apart from one report describing findings that cytosolic iron may alter the association between PCBP2 - Dicer and thus, the potential processing of miRNAs (53), further work is needed in this area.

Although kidney cancer is associated with well-studied risk factors including kidney cystic disease, obesity, aging, hemodialysis, and diabetes, one understudied risk factor is increased iron as a result of (a) engaging in occupations within iron/steel industries, (b) anemic patients undergoing frequent blood transfusions, or (c) tobacco smoking (51). A large number of iron metabolic molecules are deregulated in kidney tumors whose expression may be controlled by DNA methylation and which are correlated with poor patient survival (58). Iron levels are well-established to be markedly elevated in ccRCC, which is the predominant kidney cancer subtype that responds poorly to chemotherapeutics (1). Not only are ccRCC cells growth suppressed via use of iron chelators including DFO and DFX (52), but we have previously shown that chronic iron exposure in immortalized FTSECs deregulates expression of these 14q32 miRNAs (24). *In vivo* repeated administration of iron (ferric nitrilotriacetate (Fe-NTA)) in rats via intraperitoneal injections into their kidneys leads to elevated formation of renal cell tumors characterized by extensive genomic alterations similar to those found in humans (including chromosome 6, where 14q32 miRNA human homologs are located in the rat) (59). One reported link between iron and miRNA was demonstrated through the use of iron chelators (or targeting CD71 or FPN1) whereby cytosolic iron appeared to regulate the miRNA pathway (e.g., miRNA precursor processing) via poly(C)-binding protein 2 (PCBP2) which binds to Dicer by modulating their binding capacity (53). In support of this link, we found that chelating iron with DFO in 786-O cells resulted in significantly increased expression of the 14q32 miRNA, miR-410-3p.

Since ccRCC is a metabolic disease that is characterized by changes in the lipidome (60), it is notable that we have uncovered alterations in the iron metabolic markers following cellular treatment with LPA, a potent lipid mitogen with roles in cancer pathogenesis (61–64). Specifically, we have demonstrated that LPA alters intracellular labile iron levels and expression of iron metabolic mediators in epithelial cells including FTSECs and RPTECs. The mechanism underlying changes in iron content following LPA administration is presently unclear and requires further investigation. As shown in **Supplementary Figure 1**, we have shown that LPA treatment increases levels of CD71 (iron import receptor) and FTH1 (ferritin heavy chain 1); we propose that LPA may transcriptionally modulate their levels to increase their gene expression to thereby facilitate more iron entering cells. Furthermore, the underlying mechanism of how lysophospholipid mediators, including LPA and S1P, or iron modulates expression of 14q32 miRNAs remains unclear and is a future research direction.

miRNAs have been reported to elicit diverse cellular functions including the regulation of metastasis, invasiveness, angiogenesis, cell proliferation, and metabolism (65). From published *in silico* analyses, validated targets of the 14q32 miRNAs include those in the epithelial-mesenchymal transition (EMT) pathway, DNA damage response, and various growth factor signaling cascades (9). Expression of specific miRNAs at the 14q32 locus have been associated with reduced growth and migration in melanoma cells (43), cell cycle arrest in neuroblastoma cell lines (66), reduced colony growth in soft agar assay in a pediatric glioma cell line (67), reduced proliferation and reprogramming of metastasis target genes in a papillary thyroid cancer cell line (68) amongst others. However, the roles of 14q32 miRNAs in ccRCC remain understudied.

In the epithelium of the kidney, there exists both tight (apical surface with specific transmembrane proteins, occludin and claudins) and adherens junctions (basolateral surface with cadherins and catenins) (56). These junctions are involved in mediating cellular adhesiveness and permeability (56). With respect to cancer progression, the loss of these characteristics contributes to epithelial-mesenchymal transition (EMT) and increased motility/invasiveness (56). Although, it is interesting that we demonstrate that re-expression of a subset of 14q32 miRNAs (within subcluster A) not only reduces cell survival but also mediates alterations in claudin-1, a tight junction marker, there were no significant alterations in EMT markers. This suggests that other changes are likely needed to induce changes in EMT markers or migration, which may include alterations in subcluster B miRNAs. In a variety of cancer types, not only are 14q32 miRNAs reduced but a subset have been investigated towards their contributions to EMT. In particular, overexpression of miR-654-3p (a subcluster B miRNA) in papillary thyroid carcinoma (PTC) cells reduced migration along with decreased gene expression of EMT markers (68). Likewise, multiple studies have examined the roles of a subset of miRNAs located at the miR-379/miR-656 cluster (in subcluster B) in breast cancer; these were shown to be associated with tumor suppressing roles together with impacts on EMT (69). In laryngeal and nasopharyngeal cells, wherein miR-379 expression is reduced, overexpression of miR-379 reduced migratory capacity and hindered EMT (8). In osteosarcoma cells, miR-379 levels are reduced along with promoting metastasis (8). Although the effects of subcluster B miRNAs have yet to be investigated in ccRCC cells, EMT is highly relevant to ccRCC pathogenesis. Indeed, 25% of ccRCC patients present with advanced metastatic disease at time of diagnosis (70); regrettably, mortality of these patients with advanced stage disease is elevated (70). Expression of EMT markers is associated with poor ccRCC patient survival (70); thus, EMT is a key characteristic of ccRCC and its mechanisms underlying its deregulated expression is worthy of further study.

In addition, from our global proteomics analyses, we identified ATXN2 as a “top hit”; apart from one report regarding ATXN2 as a novel locus in chronic kidney disease (71), no other findings have been thus far reported with respect to kidney cancer. GO enrichment analysis reveals pathways associated with lipid and iron transport that are significantly affected by overexpression of the subset of 14q32 miRNAs. Additionally, IPA bioinformatics predicts a high correlation between the significantly differentiated proteins we experimentally observed to the subcluster A miRNAs and subsequent downstream affects relating to apoptosis and renal abnormalities.

From a treatment perspective, retention of kidney tissue and function are goals to improve quality of life under conditions of extensive disease wherein nephrectomy is frequently performed. Therefore, novel treatment regimens and identification of novel biomarkers for ccRCC patients would be of great benefit to hinder disease progression and recurrence along with improvements in quality of life. Further understanding of the regulation of 14q32 miRNAs in ccRCC may contribute towards this goal.

## Data availability statement

The datasets presented in this study can be found in online repositories. The mass spectrometry proteomics data have been deposited to the ProteomeXchange Consortium via the PRIDE (72) partner repository with the dataset identifier PXD036428.

## Author contributions

MN conceived, directed, and supervised project. MN, RC, JW, and SRo performed experiments. MN, JG, SS, MP, RC, PEG, AB, SRo, and SRe performed data analyses. MN, JG, MP, AB, and SS prepared figures. MN, RC, PEG, SRo, SRe prepared draft manuscript. MN, JG, SS, AB, and MP wrote final manuscript. All authors contributed to the article and approved the submitted version.

## Supplementary material

Supplementary Figure 1 LPA increases cell numbers and labile iron content in FTSECs. **(A)** Images were captured of FT194 cells treated with or without LPA 10uM (micromolar) for 2 days. Representative captured images of three independent experiments are shown. **(B)** Quantification of LPA-treated FT194 cells relative to control cells for 2 days with crystal violet. **(C)** Intracellular iron quantification in LPA-treated FT194 cells relative to control cells for 2 days. Three independent experiments were performed. **(D)** FT194 cells were treated with 10uM (micromolar) LPA for 48 hours. Protein lysates were run on SDS-PAGE gels and transferred to PVDF membranes for western analyses using the antibodies shown. Data is representative of three independent experiments. Densitometric analyses of three independent western blots is shown in the right panels.

Supplementary Figure 2 VHL overexpression reduces free iron availability in 769-P cells. VHL overexpression in 769-P cells was conducted. **(A)** Real-time PCR analysis of iron metabolic markers in 769-P cells (p=38) with VHL overexpression. **(B)** Western blotting analysis of HA tag and iron markers. This data represents the results of three independent experiments performed.

Supplementary Figure 3 Subcluster A overexpression in 769-P cells does not alter gene expression of EMT markers or migration potential. **(A)** Real-time PCR analyses of EMT markers (TWIST, SNAI1, SNAI2, ZEB1, and ZEB2) was performed (normalized to PP1A). **(B)** Migration of control and subcluster A mimic expressing cells was performed using Boyden Chambers after overnight incubation.

Supplementary Table 1 The 14q32 miRNA utilized in the TCGA heatmaps are listed.

Supplementary Table 2 The 19 identified protein targets for the miRNA subcluster A overexpressed cells are summarized.

Supplementary Table 3 The list of total quantifiable proteins with differential expressed proteins are highlighted.

Supplementary File 1 The median-fold change from normal and FDR adjusted p-values (q-values) for each miRNA presented in the **Figure 2** heatmaps are presented.

Supplementary File 2 The p-values, p-adjusted, and HR values are summarized for the survival plots shown in **Figure 2B**.

Supplementary File 3 The CI values for Kaplan Meier analyses are summarized.

Supplementary File 4 TCGA methylation data across the 14q32 region of interest is summarized.
