# Supplementary material for: Deregulated expression of the 14q32 miRNA cluster in clear cell renal cancer cells
Source: Front Oncol. 2023 Apr 17;13:1048419. doi: 10.3389/fonc.2023.1048419 (PMC10150008; doi:10.3389/fonc.2023.1048419)
Supplement: Supplementary Figure 1 — LPA increases cell numbers and labile iron content in FTSECs. (A) Images were captured of FT194 cells treated with or without LPA 10uM (micromolar) for 2 days. Representative captured images of three independent experiments are shown. (B) Quantification of LPA-treated FT194 cells relative to control cells for 2 days with crystal violet. (C) Intracellular iron quantification in LPA-treated FT194 cells relative to control cells for 2 days. Three independent experiments were performed. (D) FT194 cells were treated with 10uM (micromolar) LPA for 48 hours. Protein lysates were run on SDS-PAGE gels and transferred to PVDF membranes for western analyses using the antibodies shown. Data is representative of three independent experiments. Densitometric analyses of three independent western blots is shown in the right panels. [file Presentation_1.pptx]

## Slide 1
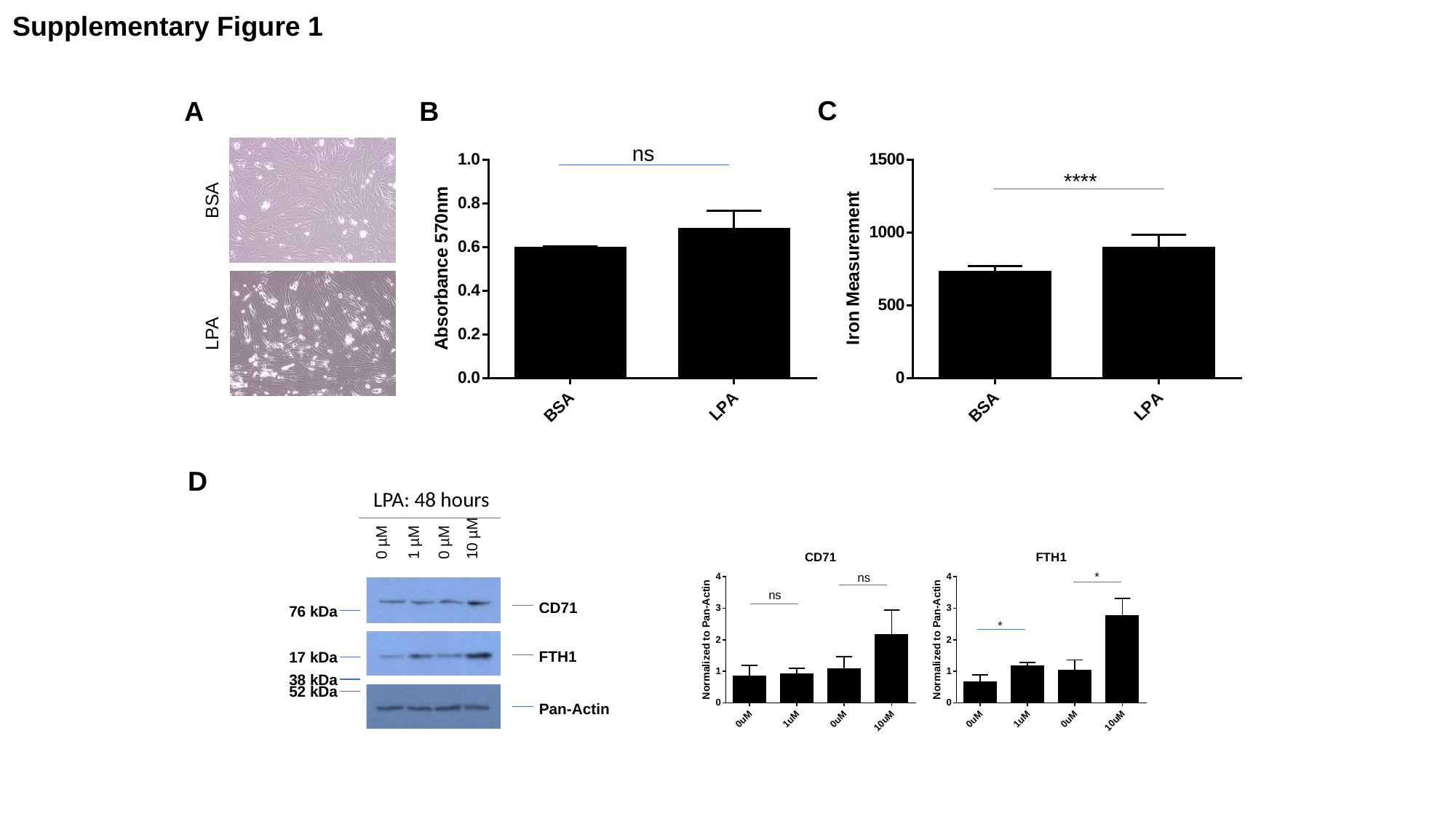

Supplementary Figure 1
C
A
B
ns
****
BSA
LPA
D
1 µM
0 µM
LPA: 48 hours
10 µM
0 µM
*
ns
ns
CD71
76 kDa
*
FTH1
17 kDa
38 kDa
52 kDa
Pan-Actin
