# Supplementary figures and images for: Deregulated expression of the 14q32 miRNA cluster in clear cell renal cancer cells
Source: Front Oncol. 2023 Apr 17;13:1048419. doi: 10.3389/fonc.2023.1048419 (PMC10150008; doi:10.3389/fonc.2023.1048419)

## Slide 1
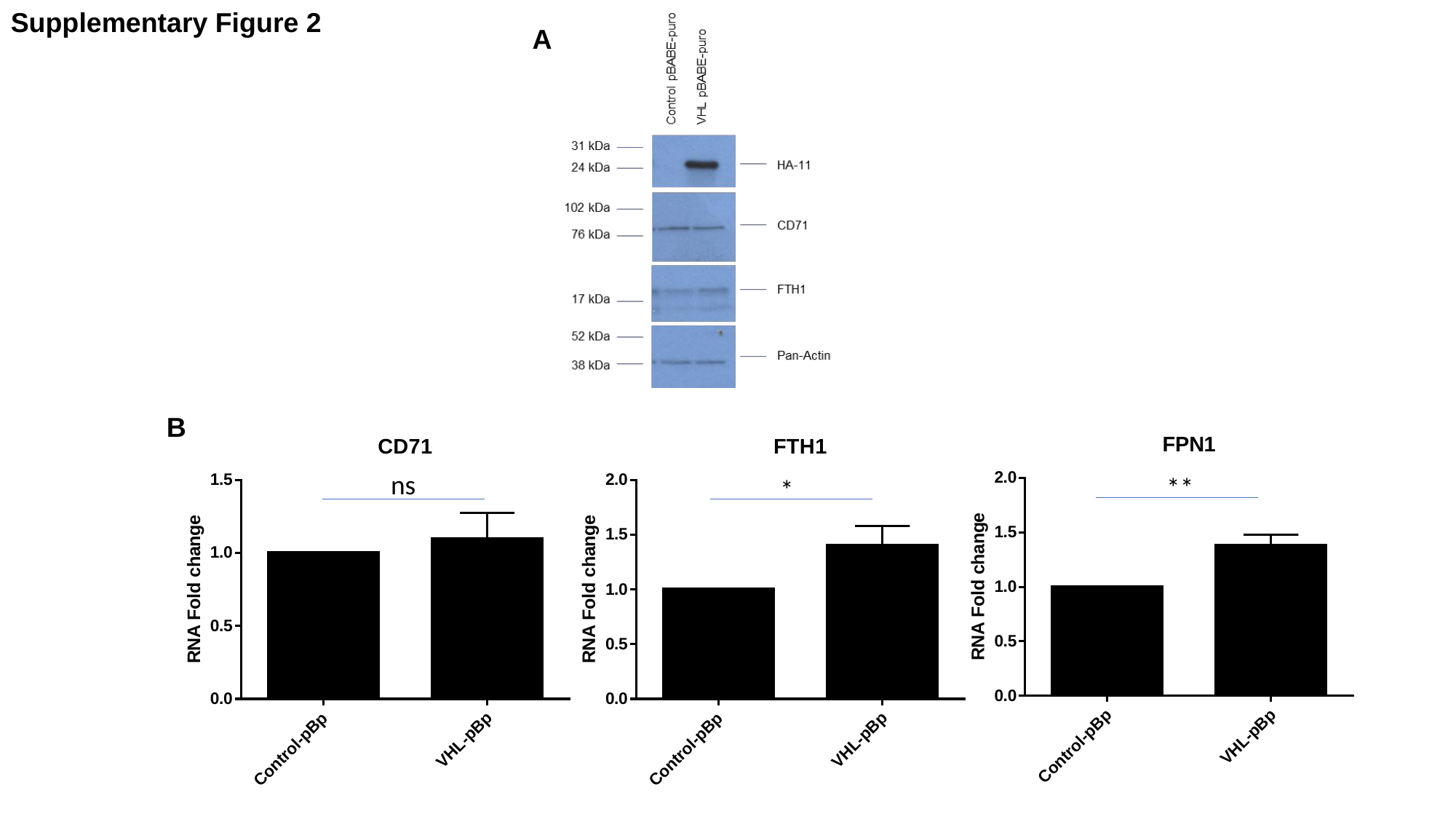

Supplementary Figure 2
A
B
ns
**
*

Supplement: Supplementary Figure 2 — VHL overexpression reduces free iron availability in 769-P cells. VHL overexpression in 769-P cells was conducted. (A) Real-time PCR analysis of iron metabolic markers in 769-P cells (p=38) with VHL overexpression. (B) Western blotting analysis of HA tag and iron markers. This data represents the results of three independent experiments performed. [file Presentation_2.pptx]
