# Supplementary material for: Deregulated expression of the 14q32 miRNA cluster in clear cell renal cancer cells
Source: Front Oncol. 2023 Apr 17;13:1048419. doi: 10.3389/fonc.2023.1048419 (PMC10150008; doi:10.3389/fonc.2023.1048419)
Supplement: Supplementary Figure 3 — Subcluster A overexpression in 769-P cells does not alter gene expression of EMT markers or migration potential. (A) Real-time PCR analyses of EMT markers (TWIST, SNAI1, SNAI2, ZEB1, and ZEB2) was performed (normalized to PP1A). (B) Migration of control and subcluster A mimic expressing cells was performed using Boyden Chambers after overnight incubation. [file Presentation_3.pptx]

## Slide 1
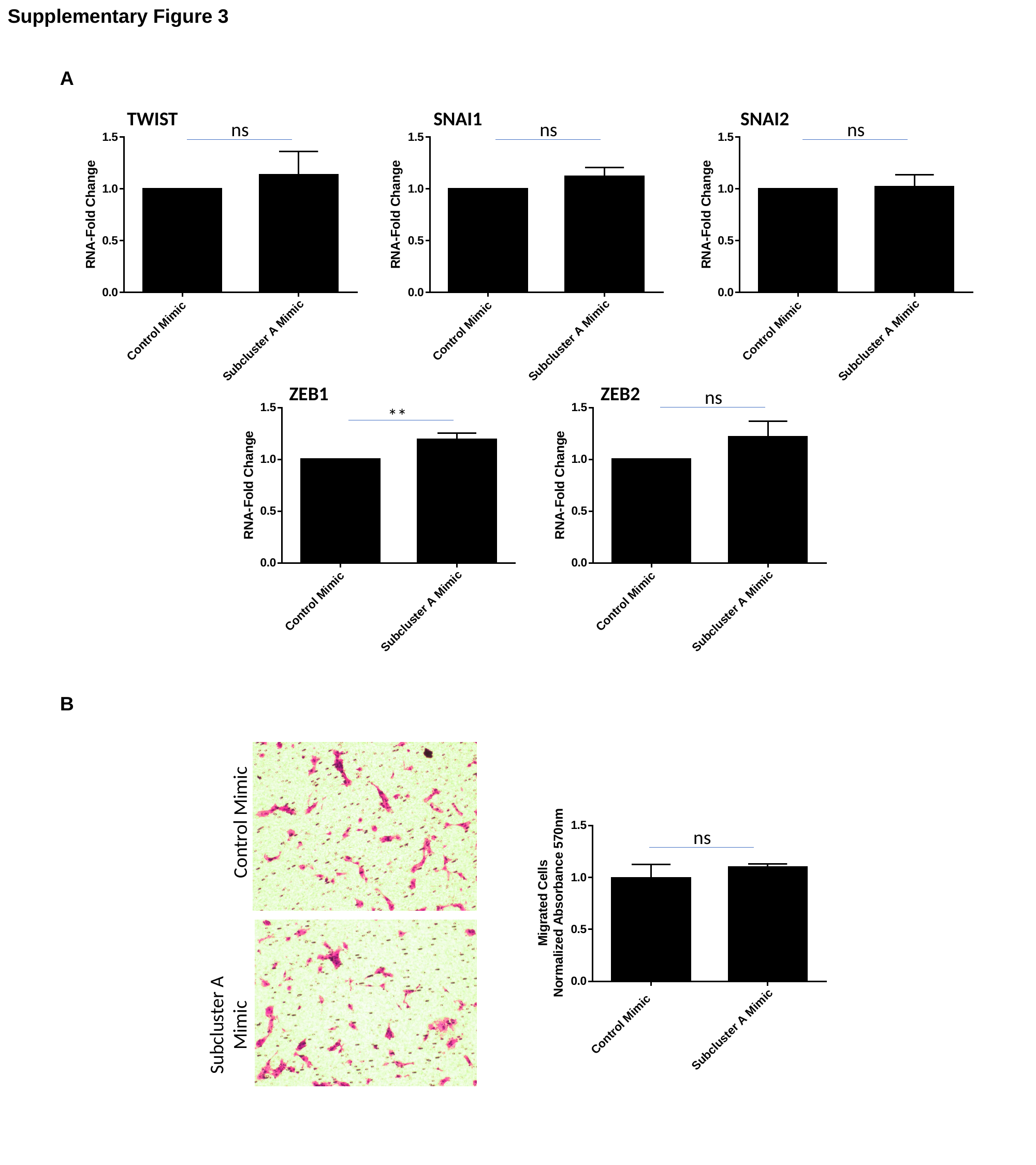

Supplementary Figure 3
A
TWIST
SNAI1
SNAI2
ns
ns
ns
ZEB1
ZEB2
ns
**
B
ns
Control Mimic
Subcluster A
Mimic
